# Beneficial Effect of Fingolimod in a Lafora Disease Mouse Model by Preventing Reactive Astrogliosis-Derived Neuroinflammation and Brain Infiltration of T-lymphocytes

**DOI:** 10.1007/s12035-023-03766-1

**Published:** 2023-11-16

**Authors:** Teresa Rubio, Ángela Campos-Rodríguez, Pascual Sanz

**Affiliations:** 1 Instituto de Biomedicina de Valencia, Consejo Superior de Investigaciones Científicas (CSIC), Jaime Roig 11, 46010 Valencia, Spain; 2 Centro de Investigación Biomédica en Red de Enfermedades Raras (CIBERER), 46010 Valencia, Spain

**Keywords:** Lafora disease, Neuroinflammation, T-lymphocyte infiltration, Fingolimod, Dimethyl fumarate

## Abstract

Lafora disease (LD; OMIM#254780) is a rare, devastating, and fatal form of progressive myoclonus epilepsy that affects young adolescents and has no treatment yet. One of the hallmarks of the disease is the accumulation of aberrant poorly branched forms of glycogen (polyglucosans, PGs) in the brain and peripheral tissues. The current hypothesis is that this accumulation is causative of the pathophysiology of the disease. Another hallmark of LD is the presence of neuroinflammation. We have recently reported the presence of reactive glia-derived neuroinflammation in LD mouse models and defined the main inflammatory pathways that operate in these mice, mainly TNF and IL-6 signaling pathways. In addition, we described the presence of infiltration of peripheral immune cells in the brain parenchyma, which could cooperate and aggravate the neuroinflammatory landscape of LD. In this work, we have checked the beneficial effect of two compounds with the capacity to ameliorate neuroinflammation and reduce leukocyte infiltration into the brain, namely fingolimod and dimethyl fumarate. Our results indicate a beneficial effect of fingolimod in reducing reactive astrogliosis-derived neuroinflammation and T-lymphocyte infiltration, which correlated with the improved behavioral performance of the treated *Epm2b-/-* mice. On the contrary, dimethyl fumarate, although it was able to reduce reactive astrogliosis, was less effective in preventing neuroinflammation and T-lymphocyte infiltration and in modifying behavioral tests.

**Supplementary Information:**

The online version contains supplementary material available at 10.1007/s12035-023-03766-1.

## Introduction

Lafora disease (LD; OMIM#254,780) is a rare, devastating, and fatal form of progressive myoclonus epilepsy. According to a recent natural history study, the disease starts in children or young adolescents and has a median time of survival of 11 years, until they die from status epilepticus and other causes, such as aspiration pneumonia and other medical complications, such as acute respiratory failure, sepsis, and SUDEP [1]. The hallmark of the disease is the accumulation of aberrant poorly branched forms of glycogen (polyglucosans, PGs) in the brain and peripheral tissues [2, 3], and the current hypothesis is that the accumulation of these PGs is causative of the pathophysiology of the disease [4]. Unfortunately, no treatment is available at the moment that could cure the disease [5]. Patients are initially treated with general anti-seizure medications, but soon, they become resistant to the action of these drugs. The disease is caused by mutations in the *EPM2A* gene [6], encoding laforin, a dual specificity phosphatase, and *EPM2B/NHLRC1* gene [7], encoding malin, an E3-ubiquitin ligase. Laforin and malin form a stable complex in which laforin recognizes specific substrates that will be ubiquitinated by malin [8, 9].

Laforin and malin are regulators of glycogen synthesis [10, 11]. They downregulate the activity of different enzymes involved in glycogen homeostasis such as glycogen synthase, glycogen phosphorylase, and the glycogenic subunits of protein phosphatase 1 (e.g., R5/PTG, R6), among others. In the absence of a functional laforin/malin complex, the synthesis of glycogen is enhanced, and probably, this is the cause of the accumulation of PGs in LD [10, 11].

LD presents alternative hallmarks such as neuroinflammation. We and others have demonstrated the presence of reactive glia in the brain of LD mice [12, 13]. Reactive astrocytes and microglia secrete pro-inflammatory mediators, which establishes a neuroinflammatory landscape that is detrimental to neuronal function [13]. Perhaps, the accumulation of PGs in the glia could trigger the expression and secretion of these pro-inflammatory mediators [14]. Recently, we have defined that the main inflammatory pathways present in LD are those that involve the TNF and IL-6 signaling pathways [15]. In addition, we defined for the first time the presence of infiltrating peripheral immune cells in the brain parenchyma of LD mice [15]. These cells could cooperate and aggravate the neuroinflammatory profile of LD.

Most of the results described above have been obtained by using LD knockout (KO) mouse models of LD with a complete loss of function of laforin (*Epm2a-/-*) [16] or malin (*Epm2b-/-*) [17]. These animals partially mimic human symptoms such as early accumulation of PGs in brain and peripheral tissues, abnormal postures of the hindlimb, memory defects, and hyperactivity with disrupted attention [16–21].

In this work, we have tested the possible benefits of two compounds with the capacity to ameliorate neuroinflammation and reduce infiltration of peripheral immune cells in the brain, using *Epm2b-/-* mice as a model of LD. On the one hand, we decided to explore the beneficial effects of fingolimod (FGD), a modulator of the sphingosine-1P receptor (S1PR) [22]. It has been reported that inhibition of S1PR in the central nervous system (CNS) with fingolimod modulates the reactivity of glial cells and ameliorates neuronal and oligodendrocyte injury, stabilizes the blood–brain barrier (BBB), and decreases peripheral immune cell recruitment into the brain parenchyma, leading to the absence of peripheral immune cells into the CNS [22]. On the other hand, we decided the use dimethyl fumarate (DMF), an oral immunomodulatory drug used in the treatment of autoimmune diseases such as multiple sclerosis [23, 24]. In addition, DMF reduces T-cell and macrophage infiltration into the spinal cord in a mouse model of experimental autoimmune encephalitis (EAE) and in multiple sclerosis patients [25, 26]. Our results confirm the beneficial effect of fingolimod by ameliorating the presence of reactive astrocyte-derived neuroinflammation and reducing the presence of infiltrating T-lymphocytes in the brain of LD mice, which correlates with a better performance in the behavioral tests.

## Materials and Methods

### Ethics Statement, Animal Care, Mice, and Husbandry

This study was carried out in strict accordance with the recommendations in the Guide for the Care and Use of Laboratory Animals of the Consejo Superior de Investigaciones Científicas (CSIC, Spain) and approved by the Consellería de Agricultura, Medio Ambiente, Cambio Climatico y Desarrollo Rural from The *Generalitat Valenciana.* All procedures were approved by the animal committee of the Instituto de Biomedicina de Valencia CSIC, (Permit number IBV-56). All efforts were made to minimize animal suffering. Male and female homozygous *Epm2b − / − *in a pure C57BL/6JRccHsd background and the corresponding control WT mice were used in this study. Mice were maintained in the IBV-CSIC facility on a 12/12 light/dark cycle under constant temperature (23 °C) with food and water provided ad libitum. When planning the experiments, the principles outlined in the ARRIVE guidelines and the Basel declaration including the 3R concept have been considered.

### Drugs and Administration

All drugs tested in this work, fingolimod (FTY-720 ref. SML0700) and dimethyl fumarate (ref. 242,926), were obtained from Sigma-Aldrich (Madrid, Spain). The treatments were performed by oral administration in drinking water at a dosage of 0.5 mg/Kg (3 µg/mL) of fingolimod and 30 mg/Kg (0.18 mg/mL) for dimethyl fumarate, per day. The drugs were dissolved in the drinking water that was used to fill the drinking bottles. Bottles were changed once a week to supply fresh compounds every single week. We measured periodically the consumption, which was similar in all the groups, control and *Epm2b-/-* mice. Three months old mice (corresponding to an early stage of LD) were treated for 15 weeks. Drug doses and administration schedules were based on a bibliographic search for both compounds [27–30]. These previous studies concluded that the doses we used in our assays were safe and that all compounds reached the brain to exert their effects. As indicated in Supplementary Fig. S1, none of the two compounds affected the progression of body weight during the treatment period, in either male or female mice. The number of mice used in each group was as follows: (1) non-treated (water) mice, 10 WT mice and 10 *Epm2b*-/-; (2) fingolimod, 16 WT mice and 16 *Epm2b*-/- mice; (3) dimethyl fumarate, 14 WT mice and 14 *Epm2b*-/- mice. In all groups, the number of male and female mice was equally represented.

### Behavioral Tests

Animals were subjected to a battery of behavioral tests conducted during the light phase. The tests started after 13 weeks of treatment, but the administration of the compounds was maintained until the end of the tests (15 weeks of treatment). The order of the behavioral tests and resting time between them were the same for each mouse. The selected battery of behavioral tests was based on the results obtained in previous reports for the use of *Epm2b-/- mice* [20, 21]. It consisted of three tests performed in the following order: hindlimb clasping, open field, and object location memory (OLM). Tests were conducted in order of increasing invasiveness: reflecting action, anxiety, and memory. Mice rested 48–72 h between tests. Behavioral tests were recorded by using the SMART Video 3.0 software from PanLab/Harvard Apparatus to evaluate mouse movement. This advanced image analysis allows the recording of activity, trajectories, and a wide variety of standard calculations related to tracking such as time/distance/entries in zones both by user-defined zones and by the entire area of mazes. We used the following tests:

1. Hindlimb clasping: Hindlimb clasping scores abnormal postures related to neurodegeneration and has been used as a marker of disease progression in a large number of neurodegenerative mouse models [31]. Mice were grasped by their tail for 10 s, and hindlimb positions were scored from 0 to 3 [32]. If the hindlimbs were consistently splayed outward, away from the abdomen, it was assigned a score of 0 (absence). If one or two hindlimbs were partially retracted toward the abdomen for more than 5 s, it received a score of 1 (mild) or 2 (moderate), respectively. If both hindlimbs were completely retracted toward the abdomen, it received a score of 3 (severe).
2. Open field: The open field test is used to assess anxiety and exploratory behaviors [33]. Mice were placed in the middle of a peripheral zone of the arena (a wall-enclosed 50 cm × 50 cm area) facing the wall and allowed to explore freely for 5 min. We analyzed the distance walked in peripheral and center areas (40% of the total surface of the area), as well as the total number of entries into the center. As anxiety levels rise, the animal tends to remain close to walls in the peripheral zone, avoiding entry into the central zone, considered more anxiogenic.
3. Object location memory (OLM): We performed an OLM probe as previously detailed in [20] to evaluate spatial recognition memory depending on the hippocampus. In brief, mice were exposed to an empty area for 10 min 24 h before training. In the training phase, two identical objects (familiar) were placed in the arena, and the mouse was allowed to explore them for 5 min. To assess short-term memory, the test was conducted 90 min after training. In the test phase, one of the familiar objects was moved to a different location (novel location), and then the mouse explored them again for 5 min. Time exploring the objects in the familiar and novel locations was measured, and the discrimination index (DI) was calculated as follows: (time exploring the novel location of the object − time exploring the familiar)/(time exploring novel + familiar) * 100. DI was used as a measure of the recognition of novel location and location memory, as in [34]. The time exploring the object in both the familiar and the novel location was defined as total activity. Animals that did not explore more than 3 s total for both objects during testing were excluded from the analysis.

### Tissue Collection and Histopathological Analyses

Animals were euthanized by cervical dislocation; brains were removed, and the left hemisphere was immediately fixed in 4% paraformaldehyde (PFA) at 4 °C overnight. Then, they were washed three times with PBS at room temperature for 10 min. Next, hemispheres were incubated for 30 min with 50% ethanol, and then they were incubated overnight in 70% ethanol. The next day, the hemispheres were dehydrated, cleared, and embedded in paraffin for histological analyses. The samples in paraffin were sagittal sectioned at 4 µm using a microtome. The presence of polyglucosans (PGs) inclusions (the hallmark of Lafora disease) in *Epm2b*-/- mice was assessed by periodic acid Schiff (PAS) staining as detailed in [20] and [21]. Six mice (3 females and 3 males) were analyzed in total per each group (WT or *Epm2b-/-* untreated and *Epm2b-/-* treated with fingolimod or dimethyl fumarate). PAS-staining photomicrographs were acquired using a Leica DM750 microscope connected to a Leica ICC50W camera with a × 40 magnification in RGB format. The number of PGs inclusions was analyzed using the image-processing package Fiji-ImageJ.

### Immunofluorescence Analyses

Brain samples derived from six individual males and females from each group of mice were analyzed by immunofluorescence to assess reactive astrocytes and microglia, as previously described in [20] and [21]. In parallel, ten individual mice per group were analyzed for T-lymphocytes infiltration as described in [15]. Sections were deparaffined, rehydrated, and incubated with NaBH (1 mg/ml) in PBS for 40 min. Then, the samples were washed in PBS, and antigen retrieval was performed for 10 min in 10 mM citrate buffer pH 6.0. in a microwave. Then, the sections were immersed in blocking buffer (1% BSA, 5% FBS, 0.2% Triton X100, in PBS) and incubated O/N at 4 °C with the primary antibodies diluted in the blocking buffer. The primary and secondary antibodies used were as follows: mouse anti-GFAP 1:500 (sigma #G3893), guinea pig anti-Iba1 (1:300, Synaptic systems #234,308), rat anti-CD3 1/100 (abcam #ab11089), rabbit anti-CD4 1/100 (abcam ab183685), and rabbit anti-CD8 1/100 (abcam ab217344). After three washes of 10 min in PBS, sections were incubated for 1 h at room temperature with the appropriate secondary antibody Alexa Fluor-conjugates (1:300 Thermo Scientific, Madrid, Spain): anti-mouse IgG Alexa Fluor 633 (#A-21046), anti-guinea pig IgG Alexa Fluor 594 (#A11076), anti-rat IgG Alexa Fluor 594 (#A-21209), and anti-rabbit IgG Alexa Fluor 488 (#A11008), diluted in blocking buffer, washed once with PBS, incubated with DAPI (Sigma, Madrid, Spain), washed twice with PBS, and mounted in Aqua-Poly/Mount (Polysciences Inc., USA).

### Image Acquisition and Analysis

Confocal images were acquired in a Confocal Spectral Leica TCS SP8 microscope (Leica, Wetzlar, Germany). For the T-lymphocytes study, pictures of the whole hippocampus were taken at × 40 objective for each area (300 µm^2^). Fifteen to twenty z stacks separated by 0.6 µm were taken per section and maximum intensity projection. Image stitching of all the images within the hippocampus was performed using the Las X (Leica) software. For the reactive astrocytes and microglia study, three pictures per section were taken in different hippocampal areas: cornus ammonis (CA1), molecular layer of CA1 plus DG (CA1-DG), and dentate gyrus (DG) at × 40 objective. The intensity signal was quantified using the image-processing package Fiji-ImageJ (NIH, Bethesda, MD, USA).

### Western Blot

Mouse brain hippocampi (from the right hemisphere) were lysed in RIPA buffer (50 mM Tris–HCl, pH 8; 150 mM NaCl; 0.5% sodium deoxycholate; 0.1% SDS; 1% Nonidet P40; 1 mM PMSF; and complete protease inhibitor cocktail (Roche, Barcelona, Spain)) for 30 min at 4 °C with occasional vortexing. The lysates were passed ten times through a 25-gauge needle in a 1-ml syringe and centrifuged at 13,000 × g for 15 min at 4 °C. Supernatants were collected and a total of 35 µg protein was subjected to SDS-PAGE and transferred onto a PVDF membrane. Membranes were blocked in 5% (w/v) nonfat milk in Tris-buffered saline (TBS-T: 50 mM Tris–HCl, 150 mM NaCl, pH 7.4; with 0.1% Tween-20) for 1 h at room temperature and incubated overnight at 4 °C with the corresponding primary antibodies: Rabbit anti-GSDM-D (Abcam. ab219800); mouse anti-P65 (Santa Cruz, sc8008); Rabbit anti-SOCS3 (Abcam, ab16030). Mouse anti-GAPDH (Santa Cruz Biotechnologies, sc-32233) or rabbit anti-actin (Sigma, A2066) was used as housekeeping antibodies. Then, membranes were probed with suitable secondary antibodies for 1 h at room temperature. Signals were obtained by chemiluminescence using ECL Prime Western Blotting Detection Reagents (Cytiva-Amersham, RPN2232) and the image reader Fuji-LAS-4000 (GE Healthcare, Barcelona, Spain). The results were analyzed using the software Image Studio Lite version 5.2 (LI-COR Biosciences, Germany). Experiments were performed on at least three individuals from each group (males and females). Results are shown as mean with SD.

### RT-qPCR Analyses

The expression of CXCL10 was measured in the samples using SYBR green-based RT-qPCR. Mouse brain hippocampi (from the right hemisphere) were lysed with TRIzol (ThermoFisher Scientific, Madrid, Spain) and RNA purified as in [15]. For each reaction, a total of 1 µg of total RNA from each hippocampus sample was reverse transcribed with the Expand Reverse Transcriptase kit (Roche, Barcelona, Spain) following the next conditions: 65 °C for 10 min, 30 °C for 10 min, 42 °C for 45 min, and hold at 4 °C. The resulting cDNA was amplified by qPCR in a total volume of 12 µl using PowerUp SYBR Green Master Mix (Applied Biosystems, Madrid, Spain) and Protector RNase Inhibitor (Roche, Barcelona, Spain). The primer sequences were those described in [13]. SYBR green-qPCR was performed under the following conditions: 95 °C for 10 min, followed by 40 cycles of 95 °C for 15 s, 60 °C for 1 min, and 60 °C to 95 °C in increments of 0.5 °C for 30 s to generate melting curves. The data were processed using StepOnePlus software version 2.3, and expression values were calculated using the comparative Ct method. Each qPCR reaction was performed on eight biological samples. The β-Actin (Actin) gene was used as the endogenous reference control to normalize target gene expression.

### Statistical Analyses

Behavioral tests were performed with all the animals indicated above per group. Histopathological experiments were performed only in the untreated control, untreated *Epm2b-/-* and treated *Epm2b-/-* with the two drugs, using at least six mice from each group. Values are mean ± standard deviation of the mean (SD). In the case of the behavioral tests, differences between the groups were analyzed by two-way ANOVA, following Tukey’s multiple comparison tests. In the case of the histopathological analyses, differences between the groups were analyzed by one-way ANOVA following Tukey’s multiple comparison tests. In both cases, we used the Graph Pad Prism version 6.0 statistical software (La Jolla, CA, USA). *P* values have been considered as *P < 0.05, **P < 0.01, ***P < 0.001, and ****P < 0.0001.

## Results

In this work, we have tested the possible beneficial effects of fingolimod (FGD) and dimethyl fumarate (DMF) on the pathophysiology of *Epm2b-/-* mice. Treatments were administered orally in male and female mice of three months of age (corresponding to an early stage of LD) for 15 weeks. As indicated in Supplementary Fig. S1, none of the two compounds affected the progression of body weight during the treatment period. After 13 weeks of treatment, we carried out a collection of behavioral tests (open field, object location memory, and hindlimb clasping), followed, at the end of the treatment, by histopathological analyses of the presence of polyglucosan inclusions, reactive glia, neuroinflammatory markers, and infiltration of T-lymphocytes in the brain of the corresponding mice.

### Hyperactivity with Disrupted Attention of Epm2b-/- Mice Is Attenuated by Treatment with Fingolimod but not with Dimethyl Fumarate

**Fig. 1:**
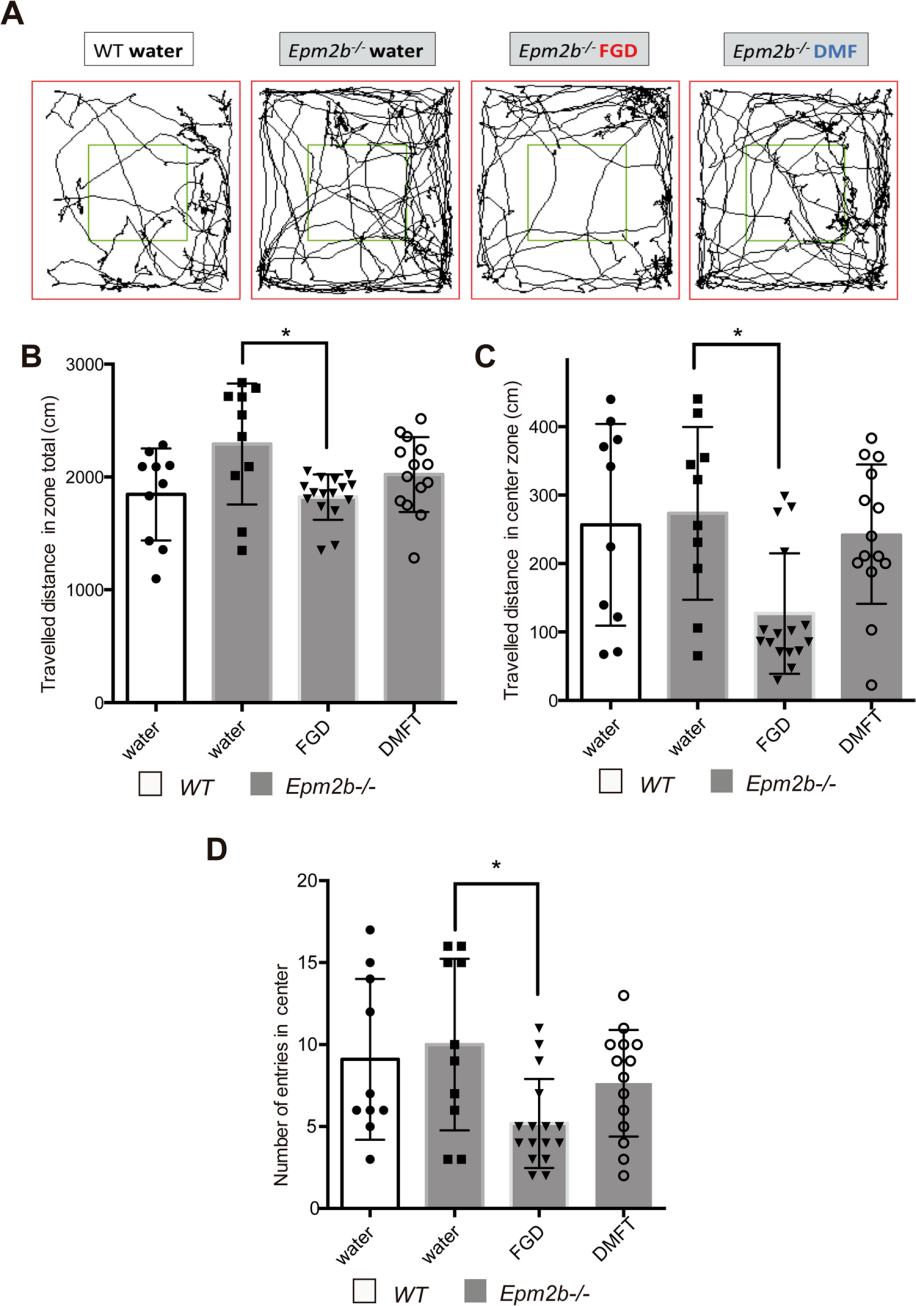
Hyperactive with disrupted attention (decreased anxiety-like) phenotype in *Epm2b-/-* and the therapeutic efficacy of fingolimod and dimethyl fumarate treatments in the open field test. **A** Representative tracks were recorded with Panlab SMART video 3.0 software to evaluate the hyperactive and anxiety-like behaviors as measured by the open field test (see Materials and Methods section). **B** Traveled distance in the peripheral area (cm), representing the hyperactivity. **C** Traveled distance in the center (cm) and **D** number of entries in the center, representing anxiety levels of each mouse. Bar graphs show the mean ± standard deviation of the mean (SD). Statistical differences between the different groups were analyzed, by two-way ANOVA following Tukey’s multiple comparison tests. *P*-values have been considered as *P < 0.05 (WT-water *n*, 10; *Epm2b-/-* water *n*, 10; *Epm2b-/-* fingolimod *n*, 16; *Epm2b-/-* dimethyl fumarate *n*, 14) (see Supplementary Table S1)

We have recently described a hyperactive with disrupted attention (decreased anxiety-like) phenotype in *Epm2b-/-* mice [20, 21]. This hyperactive phenotype was also present in the *Epm2b-/-* mice we used in this study; since as indicated in Fig. 1A and B, the traveled distance in the peripheral area of untreated *Epm2b-/-* mice showed a tendency to be higher than the one from untreated WT mice (1845.36 ± 408.02 cm vs 2291.90 ± 526.02 cm, respectively; *P* = 0.121). Treatment of *Epm2b-/-* mice with fingolimod ameliorated this phenotype: The traveled distance in the peripheral area was reduced (1821.76 ± 200.99 cm; *P* < 0.05) (Fig. 1A and B). In addition, FGD-treated mice showed a 50% reduction in the traveled distance in the center (126.97 ± 88.00 cm) in comparison to untreated *Epm2b-/-* mice (273.35 ± 126.30 cm; *P* < 0.05) (Fig. 1A and C), and the number of entries in the center was also reduced (5.18 ± 2.71 vs untreated, 10.00 ± 5.23; *P* < 0.05) (Fig. 1A and D), indicating that FGD made the *Epm2b-/-* mice more anxious. On the contrary, dimethyl fumarate treatment of *Epm2b-/-* mice had no effect in either the traveled distance in the peripheral area (2021.65 ± 331.12 cm; *P* = 0.553), the traveled distance in the center (241.45 ± 101.00 cm; *P* = 0.980) or in the number of entries in the center (7.64 ± 3.25; *P* = 0.732) in comparison to untreated *Epm2b-/-* mice (Supplementary Table S1).

### Fingolimod but not Dimethyl Fumarate Decreases Hyperactivity of Epm2b-/- Mice in the OLM Test

**Fig. 2:**
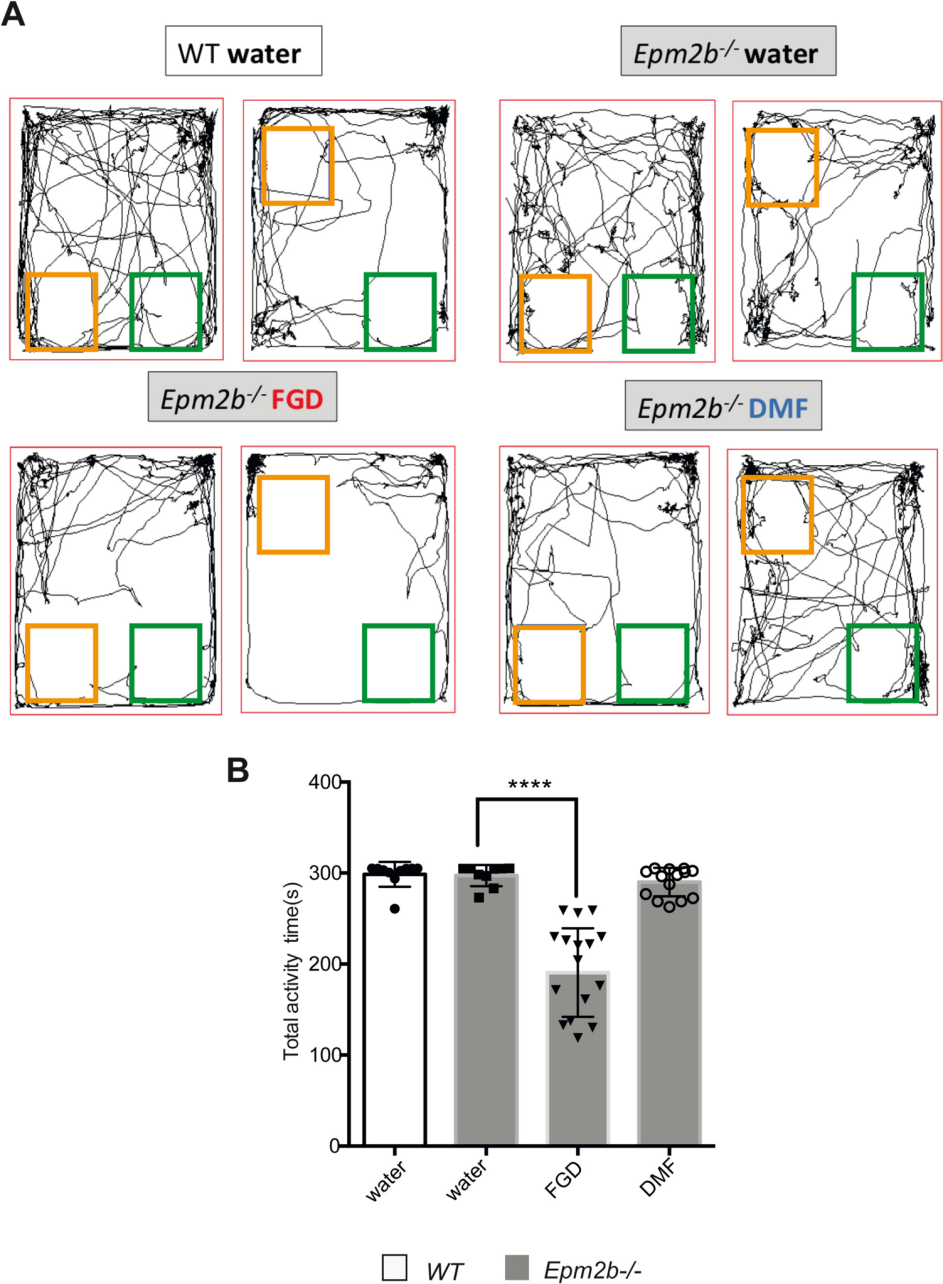
Therapeutic efficacy of fingolimod and dimethyl fumarate treatments on the object location memory test (OLM). **A** Representative tracks were recorded with Panlab SMART video 3.0 software to evaluate the spatial location memory as measured by the OLM test (see Materials and Methods section). **B** Total activity time in seconds represents the time exploring the object in both the familiar and the novel location of each mouse. Bar graphs show mean ± standard deviation (SD). Statistical differences were analyzed, by two-way ANOVA following Tukey’s multiple comparisons tests. *P*-values have been considered ****P < 0.0001 (WT-water *n*, 10; *Epm2b-/-* water *n*, 10; *Epm2b-/-* fingolimod *n*, 16; *Epm2b-/-* dimethyl fumarate *n*, 14) (see Supplementary Table S1)

The cognitive profile of *Epm2b-/-* mice was evaluated by assessing the spatial short-term memory using the object location recognition test (OLM) (Fig. 2). The discrimination index (DI) of object location and the total activity time were measured. We found no remarkable differences in DI among the groups (see Supplementary Table S1), suggesting that short-term location memory was not affected by the FGD or DMF treatments. However, we noticed a decrease in the total activity time in the *Epm2b-/-* FGD-treated mice (190.69 ± 48.69 s) in comparison to *Epm2b-/-* untreated mice (297.31 ± 11.57 s; *P* < 0.0001). The effect of fingolimod was also observed in WT controls since FGD treatment decreased their total activity time (FGD 239.46 ± 52.18 s, vs untreated 298.63 ± 13.68 s; *P* < 0.0001). On the contrary, *Epm2b-/-* DMF-treated mice did not show any statistical difference in the total activity time in comparison to untreated mice (290.13 ± 15.58 s; *P* = 0.995) (Supplementary Table S1). These results confirmed the normalization of the hyperactive behavior of *Epm2b-/-* mice described above upon the administration of FGD.

### Neurodegenerative Signs of Epm2b-/- Mice Are Ameliorated by Fingolimod and Dimethyl Fumarate

**Fig. 3:**
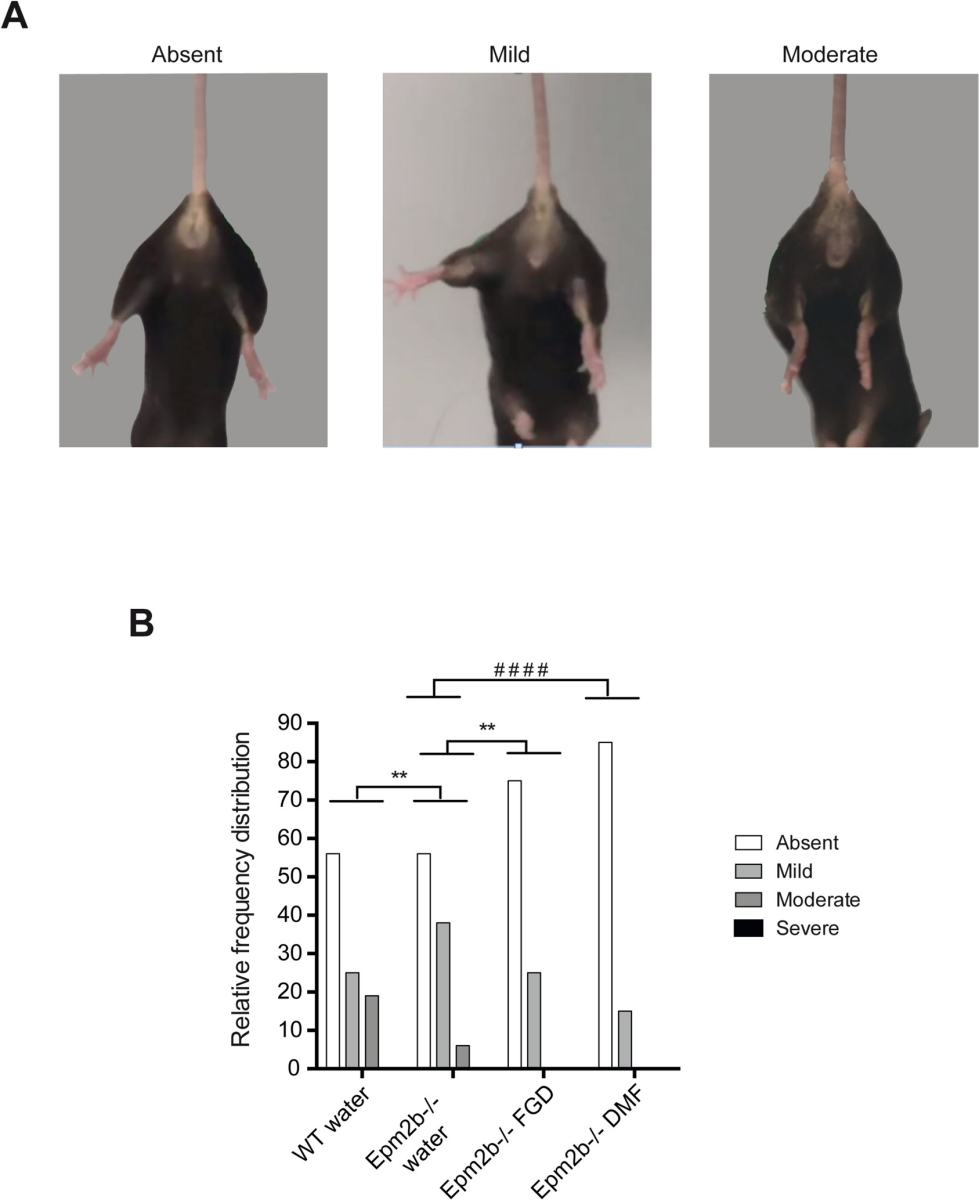
Neurodegenerative state in *Epm2b − / − *mice and the therapeutic efficacy of fingolimod and dimethyl fumarate treatments on the hindlimb clasping test. **A** Representative pictures of the three main phenotypes observed (absent, mild, and moderate). **B** Relative frequency distribution of the hindlimb clasping score representing the severity of neurodegenerative signs (see Material and Methods section). Frequency histograms show frequency distribution among 4 scores: absent, mild, moderate, and severe. Statistical differences between groups were analyzed by Pearson’s chi-square test or by Fisher’s exact test when sample sizes were zero. Statistical significance was defined as **P < 0.01 and.^####^*P* < 0.0001 (WT-water *n*, 10; *Epm2b-/-* water *n*, 10; *Epm2b-/-* fingolimod *n*, 16; *Epm2b-/-* dimethyl fumarate *n*, 14) (see Supplementary Table S1)

We also scored the neurodegenerative signs of the mice using the hindlimb clasping test. *Epm2b-/-* mice showed a worse phenotype than WT controls (*P* < 0.01; see Supplementary Table S1). In *Epm2b-/-* mice, we observed a significant improvement after the treatment with FGD (*P* < 0.01) and DMF (*P* < 0.0001) in comparison to the untreated *Epm2b-/-* mice (Fig. 3). These beneficial effects of FGD and DMF were also observed in WT control mice upon treatment (*P* < 0.0001 for FGD and *P* < 0.05 for DMF) (Supplementary Table S1).

### Polyglucosan Accumulation Is not Affected by Fingolimod or Dimethyl Fumarate Treatments

**Fig. 4:**
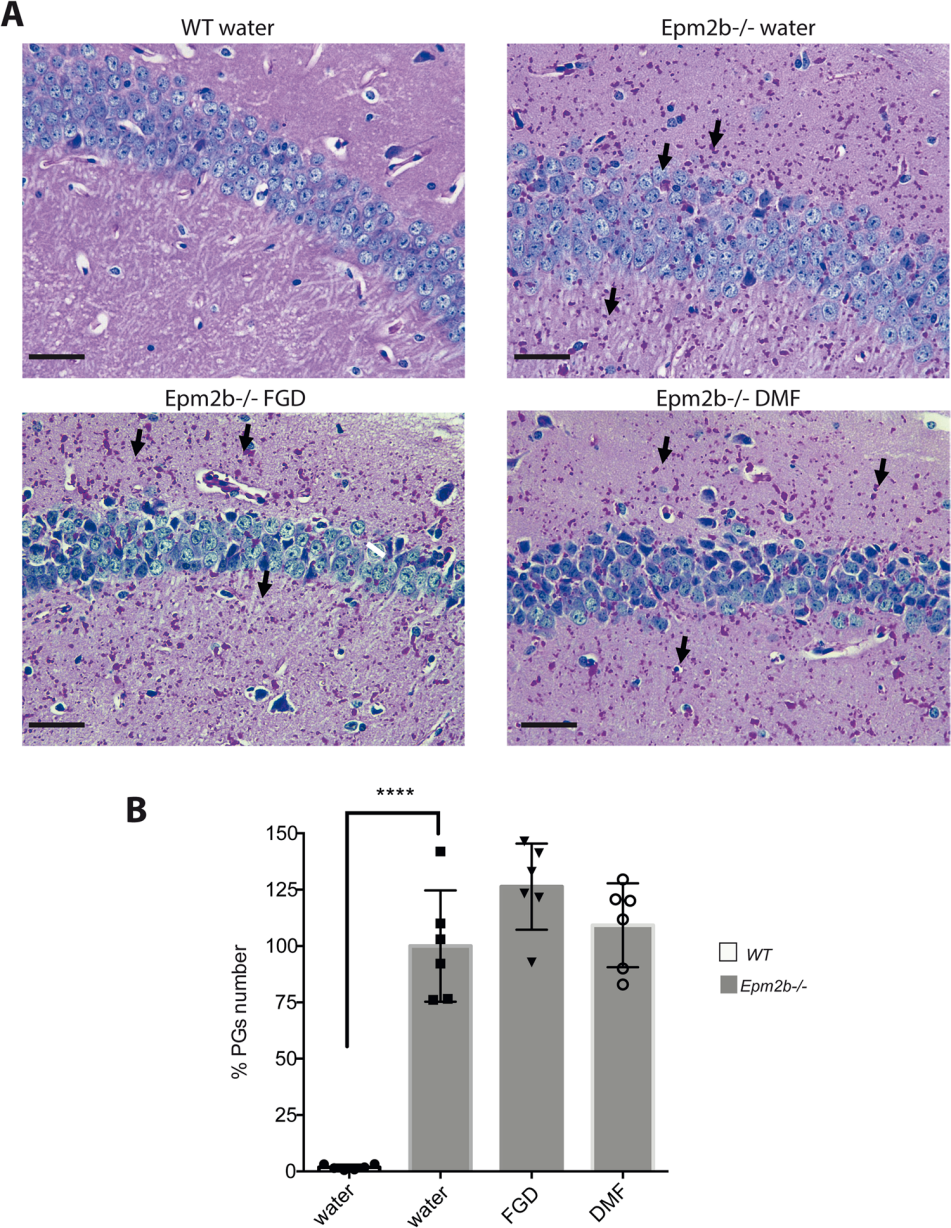
Accumulation of PGs in the hippocampus of *Epm2b − / − *mice and the therapeutic effect of fingolimod and dimethyl fumarate treatments. **A** Representative microscopy images of PGs detection (in pink; see also black arrows) in the CA1 region of the hippocampus by PAS staining; neural nuclei are in blue. The scale bar corresponds to 50 µm. **B** Percentage (%) of PG numbers versus the number present in untreated *Epm2b − / − *mice. Bar graphs show the mean ± standard deviation of the mean (SD). Statistical differences were analyzed by one-way ANOVA following Tukey’s multiple comparison tests. Statistical significance was defined as ****P < 0.0001 (*n*, 6 in all the groups) (see Supplementary Table S1)

After assessing the behavioral tests, mice were sacrificed, and we analyzed the presence of polyglucosan bodies in their brain. As it is shown in Fig. 4, untreated *Epm2b-/-* mice showed a relatively large number of polyglucosan inclusions (PGs) in comparison to WT control mice (100.00 ± 24.68 vs 1.87 ± 1.14, respectively; *P* < 0.0001), validating the characteristic phenotype of the *Epm2b-/-* mice. However, treatment with either FGD (126.36 ± 19.11; *P* = 0.093) or DMF (109.20 ± 18.60; *P* = 0.387) did not reduce the number of PGs in comparison to untreated mice (Supplementary Table S1).

### Effect of Fingolimod and Dimethyl Fumarate on the Presence of Reactive Glia

**Fig. 5:**
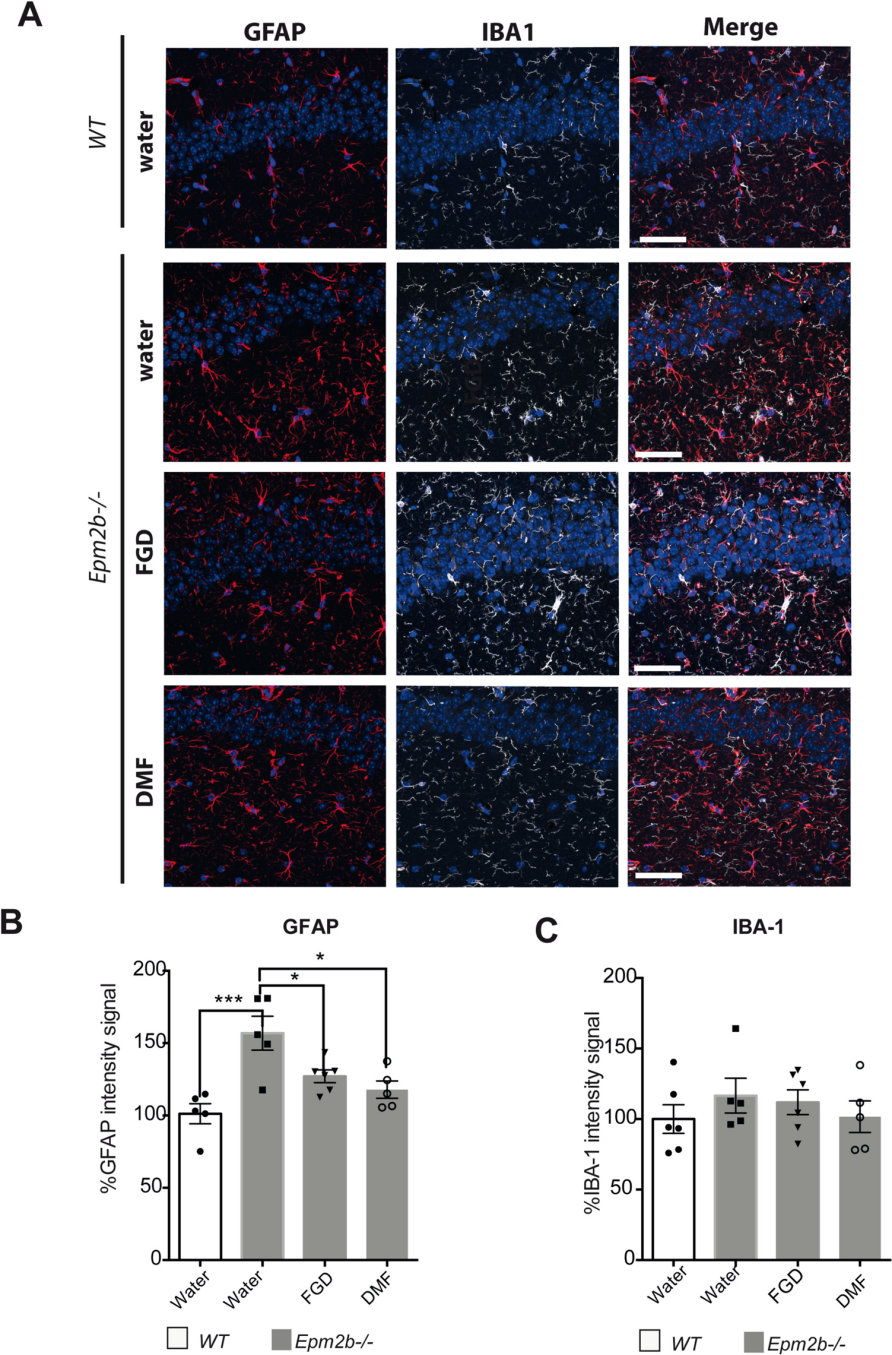
Effect of the different treatments on the presence of reactive astrocytes and microglia in the *Epm2b − / − *mice. **A** Representative immunofluorescence confocal images of the CA1 region of the hippocampus from WT control (first row) or *Epm2b-/-* mice treated with water, fingolimod, or dimethyl fumarate. Astrocytes (GFAP staining) are in red, microglia (Iba-1 staining) are in white, and DAPI staining of cellular nuclei is in blue. The scale bar corresponds to 100 µm. **B** Percentage (%) of the GFAP intensity signal related to the value obtained in untreated WT mice, representing the extension of the reactive astrogliosis in the hippocampus. **C** Percentage (%) of the Iba-1 intensity signal related to the value obtained in untreated WT mice, representing the extension of the reactive microglia in the hippocampus. Bar graphs show the mean ± standard deviation of the mean (SD). Statistical differences were analyzed by one-way ANOVA following Tukey’s multiple comparison tests. Statistical significance was defined as *P < 0.05 **P < 0.01 ***P < 0.001 (*n*, 6 in all the groups) (see Supplementary Table S1)

As we have described that *Epm2b-/-* mice are characterized by the presence of reactive glia [13], we checked the presence of this type of cells after the treatments. In agreement with previous results ([13, 20, 21]), we observed higher intensity of the reactive astrocyte marker GFAP in *Epm2b-/-* mice (156.92 ± 26.24) in comparison to WT controls (100.00 ± 15.58; *P* < 0.001). Treatment of *Epm2b-/-* mice with FGD diminished the intensity of the GFAP signal (127.09 ± 10.90; *P* < 0.05) in comparison to untreated mice, and the same was true for the DMF treatment (117.83 ± 13.38; *P* < 0.05) (Fig. 5B) (Supplementary Table S1). However, no statistically significant differences were found when we analyzed the intensity of the microglia-related signal (Iba1) among the different groups (Fig. 5C) (Supplementary Table S1).

### Effect of Fingolimod and Dimethyl Fumarate on the Presence of Several Neuroinflammatory Mediators

**Fig. 6:**
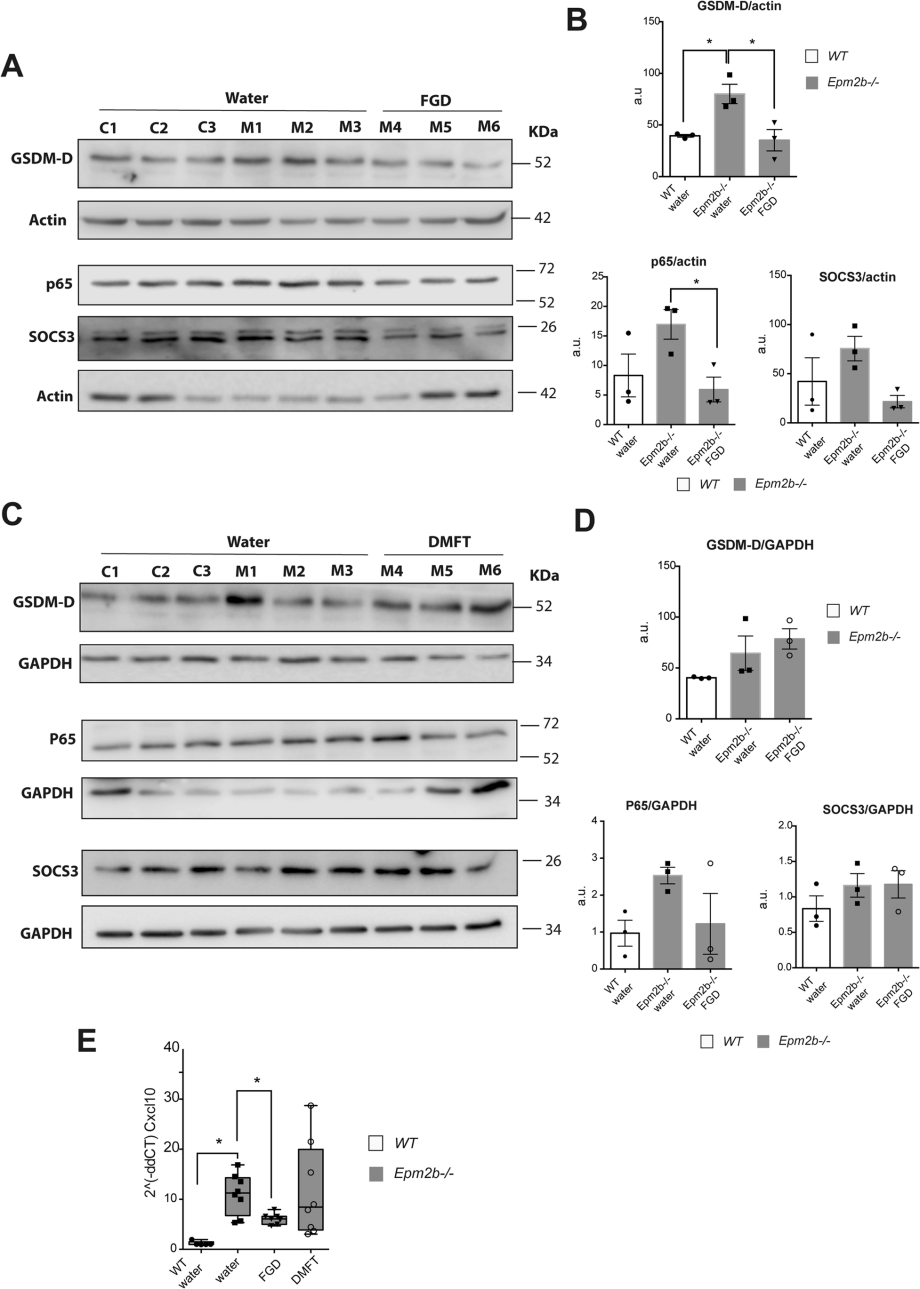
Effect of fingolimod (FGD) on the expression of different components of the inflammatory pathways activated in the *Epm2b*-/- mice. Protein levels of gasdermin-D (GSDM-D), P65/NFkB, and SOCS3 were assessed by western blot of hippocampi extracts from WT (C1-C3), untreated *Epm2b*-/- (M1-M3) and *Epm2b*-/- mice treated with fingolimod (FGD) (M4-M6) in panels (**A**), or treated with dimethyl fumarate (DMF), in panels (**C**), using the indicated antibodies. Molecular weight standards are on the right. Densitometric quantification of the corresponding blots was carried out as described in Methods; protein levels of GSDM-D, P65, and SOCS3, were related to the levels of actin and represented as arbitrary units (a.u.) for fingolimod (**B**) or related to the levels of GADPH for dimethyl fumarate (DMF) (**D**). Results are expressed as the mean ± standard deviation of the mean (SD) of three independent samples from each group. **E** CXCL10 mRNA levels were assessed by quantitative-RT-PCR. Results are expressed as mean with a range of eight independent samples from each group, referred to the values found using actin as a housekeeping gene. Differences between groups were analyzed by one-way ANOVA following Tukey’s multiple comparison tests. Statistical significance was defined as *P < 0.05 and **P < 0.01 (*n*, 8) (see Supplementary Table S1)

We have recently described that the TNF and IL6 signaling pathways are the main inflammatory pathways related to LD [15]. To analyze if the beneficial effects of FGD and DMF were related to a decrease in the presence of mediators related to these pathways, we analyzed by western blot the presence of some representative components of these pathways, such as gasdermin-D (GSDM-D), P65/NFkB, and SOCS3. As shown in Fig. 6A and B, the levels of gardermin-D were elevated in the samples from *Epm2b-/-* mice in comparison to WT controls (80.03 ± 16.26 in *Epm2b-/-* mice vs 39.49 ± 2.30 in WT control; *P* < 0.05), and the treatment with FGD decreased the levels of GSDM-D (35.23 ± 17.95; *P* < 0.05) (Fig. 6A and B). The levels of P65 and SOCS3 also showed a tendency to be higher in untreated *Epm2b-/-* samples in comparison to WT (Supplementary Table S1), indicating that around 7 months of age, *Epm2b-/-* mice start showing an increase in these inflammatory markers. In *Epm2b-/-* mice, FGD-treatment was also effective in decreasing the levels of P65/NFkB (5.92 ± 3.63 vs untreated 16.96 ± 4.36; *P* < 0.05) (Fig. 6A and B) and SOCS3 (21.57 ± 10.91 vs untreated 75.61 ± 21.52; *P* < 0.05) in comparison to untreated mice (Fig. 6A and B). On the contrary, treatment with DMF did not statistically modify the levels of these mediators in comparison to untreated *Epm2b-/-* mice (Fig. 6C and D; Supplementary Table S1). In addition, we analyzed the expression of the chemokine CXCL10, as it is one of the early components of the inflammatory process present in LD [13]. In agreement with previous results [13], the expression of CXCL10 was higher in *Epm2b-/-* mice (11.06 ± 4.05) in comparison to WT controls (1.19 ± 0.42; *P* < 0.01) (Fig. 6E). Interestingly, the expression of this chemokine was decreased upon the treatment with FGD (6.11 ± 1.09; *P* < 0.05). On the contrary, DMF did not modify the expression of CXCL10 (11.69 ± 9.35; *P* = 0.629) (Fig. 6E) (Supplementary Table S1). Therefore, at around 7 months of age, *Epm2b-/-* mice present some clear signs of inflammation which are ameliorated by FGD treatment.

### Effect of Fingolimod and Dimethyl Fumarate on the Infiltration of T-lymphocytes

**Fig. 7:**
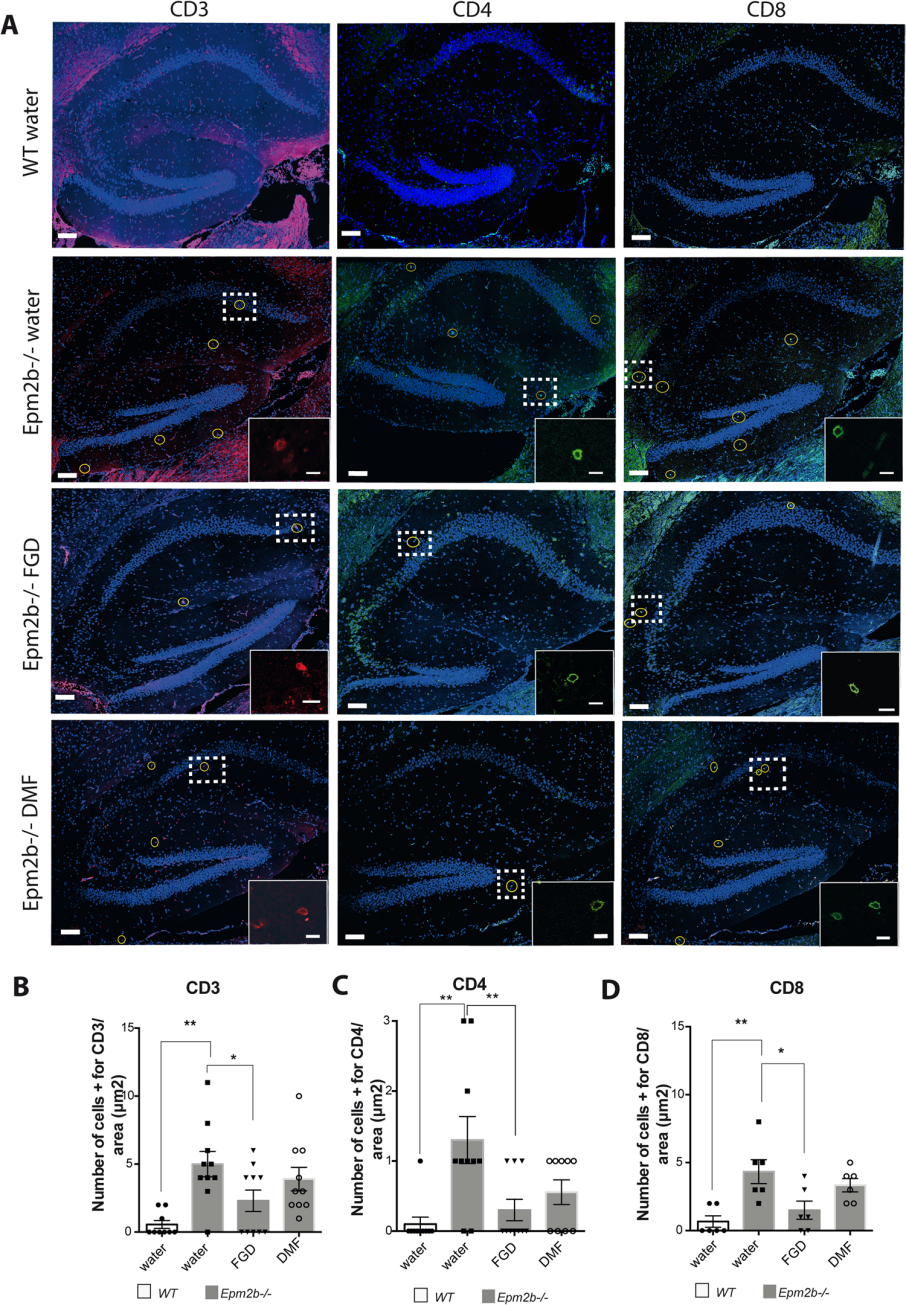
Effect of the different treatments on the T-lymphocytes infiltration in the hippocampus of the *Epm2b*-/- mice. **A** Representative confocal images of the whole hippocampus from untreated WT mice or *Epm2b*-/- mice untreated (water) or treated with fingolimod or dimethyl fumarate, stained with rat-anti CD3 (T-cell marker in red, first column) in combination with mouse-anti CD4 (helper T-cell marker in green, middle column) or rabbit-anti CD8 (cytotoxic T-cell marker in green, third column). The scale bar corresponds to 100 µm. A squared dashed line indicates the area that is magnified (scale bar 10 µm). The CD3 + , CD4 + , or CD8 + cells were selected by morphology following the criteria shown in the magnified image. Oval yellow marks the T-lymphocytes in the full hippocampus. **B–D** Quantification of the number of infiltrating cells in the hippocampus of each group of mice in an area of 300 µm.^2^. **B** CD3 + cells. **C** CD4 + cells. **D** CD8 + cells. Bar graphs show the mean ± standard deviation of the mean (SD). The differences between groups were analyzed by one-way ANOVA following Tukey’s multiple comparison tests. Statistical significance was defined as *P < 0.05 and **P < 0.01 (depending on the groups, from 6 to more than 10 samples were analyzed) (see Supplementary Table S1)

We have also recently described that there is an infiltration of T-lymphocytes in the brain parenchyma of *Epm2b-/-* mice, which could aggravate the neuroinflammatory phenotype present in these mice [15]. So, we analyzed whether the FGD and DMF treatments could affect this infiltration. As shown in Fig. 7, at 6 months of age, the *Epm2b-/-* mice already showed signs of T-lymphocyte infiltration, since the number of CD3 + , CD4 + , and CD8 + T-lymphocytes in an area of 300 µm^2^ of the hippocampus was significantly higher (CD3 + , 5.0 ± 2.9; CD4 + , 1.3 ± 1.1; CD8 + , 4.3 ± 2.2) than in the WT controls (CD3 + , 0.5 ± 0.5; CD4 + , 0.1 ± 0.3; CD8 + , 0.7 ± 1.0; *P* < 0.01 in all the cases) (Fig. 7A–D). Treatment of *Epm2b-/-* mice with FGD reduced the number of infiltrated T-cells in comparison to untreated mice (CD3 + , 2.3 ± 2.4, *P* < 0.05; CD4 + , 0.3 ± 0.5, *P* < 0.01; CD8 + , 1.5 ± 1.6, *P* < 0.05) (Fig. 7A–D). However, DMF treatment, although it showed a tendency to decrease the number of infiltrated T-lymphocytes (CD3 + , 3.9 ± 2.7; CD4 + , 0.6 ± 0.5; CD8 + , 3.3 ± 1.2), the results were not statistically significant (*P* > 0.119) (Fig. 7A–D) (Supplementary Table S1).

## Discussion

Lafora disease (LD) is a rare, devastating, and fatal neurological disorder that has no treatment yet. As one of the hallmarks of the disease is the accumulation of polyglucosan inclusions (PGs) in the brain and peripheral tissues, several groups have initiated different strategies to prevent/ diminish the accumulation of the PGs. The main therapeutic target in these approaches has been the glycogen synthase isoform present in the brain (GYS1). The expression of this gene has been diminished either by the use of specific antisense oligonucleotides [35], microRNA [36], or CRISPR/Cas9 approaches [37]. Alternatively, small molecule-specific inhibitors of GYS1 have been obtained [38]. Another alternative has been the use of antibody-fusion enzymes to degrade the PGs in the brain (e.g., alpha-amylase based VAL0417, alpha-glucosidase based VAL1221) [39]. However, although these approaches have been validated in LD animal models, to our knowledge, none of them has reached the clinic yet.

An alternative approach to develop possible treatments for LD has been the use of repurposing drugs to tackle another hallmark of LD, namely neuroinflammation [13, 15]. Some years ago, our group defined the beneficial effect of metformin on LD mouse models [40], and this allowed the designation of metformin as an orphan drug for the treatment of LD by the European Medicines Agency (EMA) and the American Federal Drug Administration (FDA). Metformin has also been used to treat patients, and recent reports indicate a beneficial effect of this compound by slowing down the progression of the disease [41]. To provide an alternative to the use of metformin, our group has checked the possible beneficial effect of alternative repurposing drugs. We recently reported the beneficial effect of propranolol [20] and memantine and minocycline [21].

Recently, our group has reported on the main inflammatory pathways present in LD mice, and our results suggest that the TNF and IL-6 signaling pathways are the main responsible inflammatory mediators present in LD [15]. In addition, we defined, for the first time, the presence of infiltrating T-lymphocytes in the brain parenchyma of LD mice, which could aggravate the neuroinflammation present in LD [15]. To prevent the infiltration of peripheral immune cells, in this work, we decided to use fingolimod (FGD) and dimethyl fumarate (DMF).

Fingolimod is a disease-modifying drug with oral immunosuppressant effects that has been approved for the treatment of multiple sclerosis by the FDA in 2010. At the level of the central nervous system (CNS), modulators (antagonists) of S1PR1 signaling, like fingolimod, have specific actions on every CNS cell type: (i) in endothelial cells, by reducing the permeability of the blood–brain barrier (BBB), decreasing expression of ICAM-1, and reducing the binding of leukocytes to endothelial cells; (ii) in neurons, by preventing apoptosis and protecting from excitotoxic death; (iii) in astrocytes, by inhibiting the production of proinflammatory cytokines, chemokines, and neurotoxic substances (e.g., IL-6, COX2, VEGF), by increasing the production of neuroprotective factors and by preventing the activation and proliferation of astrocytes; (iv) in microglia, by reducing microglia activation, reducing the production of proinflammatory cytokines (IL-6), and by promoting polarization to M2 phenotype; (v) in oligodendrocytes, by promoting the renewal of oligodendrocytes and enhancing remyelination. In conclusion, fingolimod reduces inflammation, excitotoxicity, glial activation and polarization, and BBB destruction, and improves neurogenesis [42]. For all these reasons, fingolimod has a beneficial effect on different neurological disorders such as stroke [42], hypoxia [43], and epilepsy [44–47].

In this work, we demonstrate that fingolimod had also beneficial effects on a mouse model of Lafora disease (LD). It prevented the activation of astrocytes (lower levels of GFAP marker) and decreased the levels of different components of the TNF and IL6 inflammatory signaling pathways present in LD, such as gasdermin-D and CXCL10. In untreated *Epm2b-/-* mice, the levels of P65 and SOCS3 also showed a tendency to be higher than in WT (Supplementary Table S1), indicating that around 7 months of age, *Epm2b-/-* mice started showing an increase in these inflammatory markers. Fingolimod also decreased the levels of these markers in *Epm2b-/-* mice. In addition, it diminished the number of peripheral T-lymphocytes in the brain parenchyma (lower levels of CD3 + , CD4 + , and CD8 + cells). Probably, this could be the reason for the observed beneficial effects in the behavioral tests of the treated animals: amelioration of the hyperactivity in the open field and in the OLM tests and amelioration in the neurodegenerative signs in the hindlimb clasping test (Table 1).

**Table 1:** Summary of the beneficial effect of fingolimod and dimethyl fumarate on *Epm2b-/-* mice. ^a^Respect to WT control in water; ^b^Respect to *Epm2b-/-* in water. Statistical significance is considered *P < 0.05, **P < 0.01, ***P < 0.001, ****P < 0.0001 (see Supplementary Table S1). In bold we emphasize the differences between the groups

Behavioral or histological features Corresponding parameter	*Epm2b*-/- water^a^	*Epm2b*-/- fingolimod^b^	*Epm2b*-/- dimethyl fumarate^b^
**Hyperactivity** Open field: traveled distance in the peripheral area (cm)	Not modified *P* = 0.121	**Ameliorated** *P* < 0.05	Not modified *P* = 0.553
**Anxiety** Open field: traveled distance in the center (cm)	Not modified *P* = 0.993	**Increased** *P* < 0.05	Not modified *P* = 0.980
**Hyperactivity** OLM: total activity time (s)	Not modified *P* = 0.999	**Ameliorated** *P* < 0.0001	Not modified *P* = 0.995
**Neurodegenerative signs** Hindlimb clasping score	**Increased** *P* < 0.01	**Ameliorated** *P* < 0.01	**Ameliorated** *P* < 0.0001
**PG inclusions PAS** % LBs	**Increased** *P* < 0.0001	Not modified *P* = 0.093	Not modified *P* = 0.387
**Astrogliosis** % GFAP intensity signal	**Increased** *P* < 0.001	**Ameliorated** *P* < 0.05	**Ameliorated** *P* < 0.05
**Microgliosis** % IBA-1 intensity signal	Not modified *P* = 0.247	Not modified *P* = 0.900	Not modified *P* = 0.532
**GSDM-D protein levels**	**Increased** *P* < 0.05	**Ameliorated** *P* < 0.05	Not modified *P* = 0.199
**P65/NFkB protein levels**	Not modified *P* = 0.200	**Ameliorated** *P* < 0.05	Not modified *P* = 0.700
**SOCS3 protein levels**	Not modified *P* = 0.200	**Ameliorated** *P* < 0.05	Not modified *P* = 0.900
**CXCL10 RNA levels**	**Increased** *P* < 0.01	**Ameliorated** *P* < 0.05	Not modified *P* = 0.629
**T-lymphocyte infiltration** Number of T-cells in 300 µm^2^	**Increased** *P* < 0.01	**Ameliorated** *P* < 0.05	Not modified *P* > 0.119

We also studied the possible beneficial effects of dimethyl fumarate. DMF is an oral immunomodulatory drug used in the treatment of autoimmune diseases such as multiple sclerosis. It received the FDA-approval in 2013, but its mechanism of action is still poorly understood. It has been shown that DMF activates the nuclear factor erythroid 2-related factor (Nrf2), having antioxidant effects [23, 24]. In addition, DMF reduces T-cell and macrophage infiltration into the spinal cord in a mouse model of experimental autoimmune encephalitis (EAE) and in multiple sclerosis patients [25, 26]. Recently, it has been described that DMF reduced microglial activation (Iba1) in the short term and reduced the infiltration of CD4 + and CD8 + T-lymphocytes in the brain of a rat model of EAE [29]. DMF also acts as a strong agonist of HCAR2, a G protein-coupled membrane receptor expressed in immune cells, inducing robust anti-inflammatory signaling. DMF also prevents microglia activation and the production of pro-inflammatory mediators, possibly explaining its beneficial effects in alleviating seizures in a pentylenetetrazole (PTZ)-induced rat model [23, 48].

Consistent with the prevention of glia activation defined above, DMF prevented the activation of astrocytes in LD mice (Table 1). On the contrary, we did not observe any effect in terms of decreasing the levels of different components of the TNF and IL6 inflammatory signaling pathways or preventing T-lymphocyte infiltration. Perhaps, this is the reason for the poor performance of DMF in improving behavioral tests.

In summary, this work demonstrates the beneficial effect of fingolimod in different behavioral and histopathological analyses of *Epm2b-/-* mice. We propose that fingolimod could be used in the notion of “network pharmacology” that has been formulated recently, where the design of chosen repurposing drug cocktails could be used as anti-seizure medications since it has been reported that the combined action of different repurposing drugs is more beneficial than the action of a single compound [45]. Perhaps, the use of fingolimod in combination with other repurposing drugs which have a positive effect on LD, such as metformin, propranolol, and/or memantine, could have a synergic beneficial effect on LD pathophysiology.

### Supplementary Information

Below is the link to the electronic supplementary material.

Supplementary file1 (PDF 159 KB)

## Data Availability

All data generated or analyzed during this study are included in this published article and its supplementary information files.
