# Supplementary material for: Beneficial Effect of Fingolimod in a Lafora Disease Mouse Model by Preventing Reactive Astrogliosis-Derived Neuroinflammation and Brain Infiltration of T-lymphocytes
Source: Mol Neurobiol. 2023 Nov 16;61(6):3105–20. doi: 10.1007/s12035-023-03766-1 (PMC11087365; doi:10.1007/s12035-023-03766-1)
Supplement: Supplementary file 1 — Supplementary file1 (PDF 159 KB) [file 12035_2023_3766_MOESM1_ESM.pdf]

Supplementary Material

Supplementary Fig. S1. Fingolimod and dimetylfumarate did not affect the body weight of the mice during the treatment period. Average weight in grams (g) for female **(A)** or male **(B)** mice is represented as mean ± standard deviation of the mean (SD) for each group of mice (WT in water, *Epm2b-/-* in water, fingolimod or dimetylfumarate) for each time point (weeks of treatment)**.**

Supplementary Figure S1

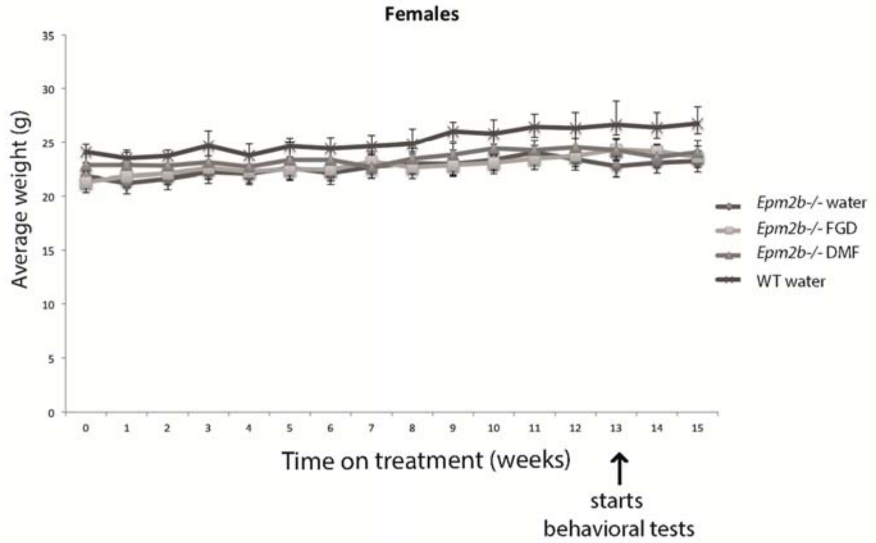

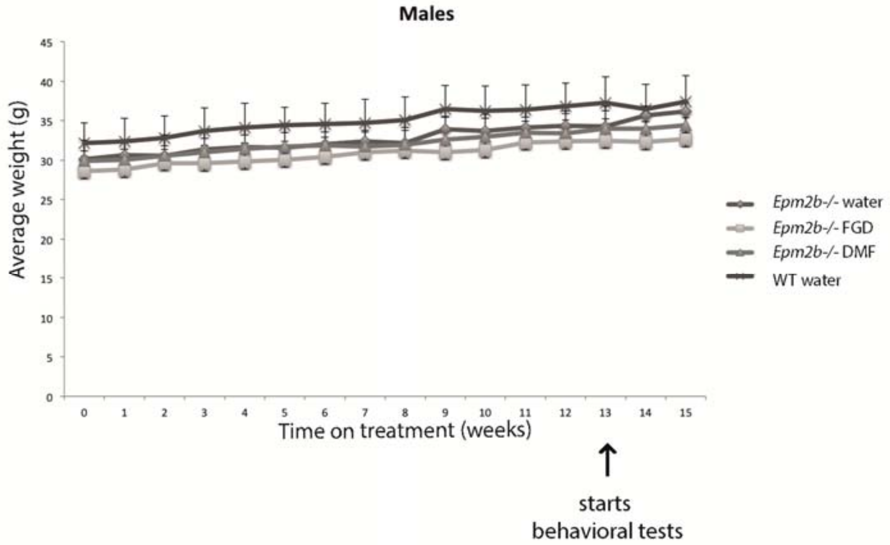

Supplementary Table S1: Statistical summary table of behavioral and histopathological variables analyzed throughout this study. The table details the behavioral test (analyzed by two-way ANOVA) or histological analysis (analyzed by one-way ANOVA) with the variable name and the mean ± SD for each variable with the significance (P-value). (a) P-values of *Epm2b-/-* variables respect to the WT control, both in water; (b) P-values of variables from treated mice respect to the corresponding mice group treated with water. nd: not determined.

Variable	Genotype	Water[a]	Fingolimod	P-value FGD[b]	Dimethyl Fumarate	P-value DMF[b]
Openfield Traveled distance in the peripheral area (cm)	WT	1845.36 ± 408.02	2275.25 ± 324.34	P=0.106	1980.49 ± 557.66	P=0.970
Openfield Traveled distance in the peripheral area (cm)	Epm2b-/-	2291.90 ± 526.02<br>P=0.121	1821.76 ± 200.99	P=0.043	2021.65 ± 331.12	P=0.553
Openfield Travelled distance in center (cm)	WT	256.59 ± 157.31	196.07 ± 85.60	P=0.731	355.66 ± 110.59	P=0.227
Openfield Travelled distance in center (cm)	Epm2b-/-	273.35 ± 126.30<br>P=0.993	126.97 ± 88.00	P=0.015	241.45 ± 101.00	P=0.980
Openfield Number entries in center	WT	9.10 ± 4.91	8.00 ± 3.43	P=0.985	11.87 ± 5.12	P=0.574
Openfield Number entries in center	Epm2b-/-	10.00 ± 5.23<br>P=0.996	5.18 ± 2.71	P=0.049	7.64 ± 3.25	P=0.732
OLM Discrimination index	WT	5.76 ± 5.59	34.21 ± 27.08	P=0.946	18.20 ± 31.04	P=0.998
OLM Discrimination index	Epm2b-/-	19.77 ± 37.92<br>P=0.998	6.77 ± 20.13	P=0.998	1.57 ± 15.05	P=0.999
OLM Activity time (s)	WT	298.63 ± 13.68	239.46 ± 52.18	P<0.0001	301.01 ± 6.43	P=0.999
OLM Activity time (s)	Epm2b-/-	297.31 ± 11.57<br>P=0.999	190.69 ± 48.69	P<0.0001	290.13 ± 15.58	P=0.995
Neurodegenerative signs Hindlimb clasping (Absent, Mild,	WT	56, 25, 19, 0	90, 5, 5, 0	P<0.0001	73, 20, 7, 0	P<0.05

Moderate, Severe)						
Neurodegenerative signs Hindlimb clasping (Absent, Mild, Moderate, Severe)	Epm2b-/-	56, 38, 6, 0<br>P<0.01	75, 25, 0, 0	P<0.01	85, 15, 0, 0	P<0.0001
PGs inclusions %PG numbers vs Epm2b-/- water	WT	1.87 ± 1.14	nd	nd	nd	nd
PGs inclusions %PG numbers vs Epm2b-/- water	Epm2b-/-	100.00 ± 24.68<br>P<0.0001	126.36 ± 19.11	P=0.093	109.20 ± 18.60	P=0.387
Astrogliosis % GFAP intensity signal	WT	100.00 ± 15.58	nd	nd	nd	nd
Astrogliosis % GFAP intensity signal	Epm2b-/-	156.92 ± 26.24<br>P<0.001	127.09 ± 10.90	P<0.05	117.83 ± 13.38	P<0.05
Microgliosis % IBA1 intensity signal	WT	100.00 ± 24.75	nd	nd	nd	nd
Microgliosis % IBA1 intensity signal	Epm2b-/-	116.65 ± 27.59<br>P=0.247	111.91 ± 21.48	P=0.900	101.65 ± 25.03	P=0.532
Relative gasdermin-D levels/Actin	WT	39.49 ± 2.30	nd	nd	nd	nd
Relative gasdermin-D levels/Actin	Epm2b-/-	80.03 ±16.26<br>P<0.05	35.23 ± 17.95	P<0.05		
Relative gasdermin-D levels/GAPDH	WT	40.40 ± 0.87	nd	nd	nd	nd
Relative gasdermin-D levels/GAPDH	Epm2b-/-	64.46 ± 29.48<br>P<0.05			78.54 ± 17.37	P=0.199
Relative P65/NFkB levels/Actin	WT	8.31 ± 6.26	nd	nd	nd	nd
Relative P65/NFkB levels/Actin	Epm2b-/-	16.96 ± 4.36<br>P=0.200	5.92 ± 3.63	P<0.05		
Relative P65/NFkB levels/GAPDH	WT	0.96 ± 0.61	nd	nd	nd	nd
Relative P65/NFkB levels/GAPDH	Epm2b-/-	2.53 ± 0.39			1.22 ± 1.42	P=0.700

		P=0.200				
Relative SOCS3 levels/Actin	WT	42.14 ± 41.76	nd	nd	nd	nd
Relative SOCS3 levels/Actin	Epm2b-/-	75.61 ± 21.52<br><br>P=0.200	21.57 ± 10.91	P<0.05		
Relative SOCS3 levels/GAPDH	WT	0.83 ± 0.31	nd	nd	nd	nd
Relative SOCS3 levels/GAPDH	Epm2b-/-	1.16 ± 0.29<br><br>P=0.200			1.17 ± 0.33	P=0.900
Relative CXCL10 expression	WT	1.19 ± 0.42	nd	nd	nd	nd
Relative CXCL10 expression	Epm2b-/-	11.06 ± 4.05<br><br>P<0.01	6.11 ± 1.09	P<0.05	11.69 ± 9.35	P=0.629
T-Lymphocyte infiltration Number of cells in 300µm^2	WT	CD3 0.6 ± 0.8<br>CD4 0.1 ± 0.3<br>CD8 0.7 ± 1.0	nd	nd	nd	nd
T-Lymphocyte infiltration Number of cells in 300µm^2	Epm2b-/-	CD3 5.0 ± 2.9<br>CD4 1.3 ± 1.0<br>CD8 4.3 ± 2.0<br><br>CD3 P<0.01<br>CD4 P<0.01<br>CD8 P<0.01	CD3 2.3 ± 2.3<br>CD4 0.3 ± 0.5<br>CD8 1.5 ± 1.6	P<0.05<br>P<0.01<br>P<0.05	CD3 3.9 ± 2.7<br>CD4 0.6 ± 0.5<br>CD8 3.3 ± 1.2	P=0.252<br>P=0.119<br>P=0.145
